# Development of machine learning models for detection of vision threatening Behçet’s disease (BD) using Egyptian College of Rheumatology (ECR)–BD cohort

**DOI:** 10.1186/s12911-023-02130-6

**Published:** 2023-02-17

**Authors:** Nevin Hammam, Ali Bakhiet, Eiman Abd El-Latif, Iman I. El-Gazzar, Nermeen Samy, Rasha A. Abdel Noor, Emad El-Shebeiny, Amany R. El-Najjar, Nahla N. Eesa, Mohamed N. Salem, Soha E. Ibrahim, Dina F. El-Essawi, Ahmed M. Elsaman, Hanan M. Fathi, Rehab A. Sallam, Rawhya R. El Shereef, Faten Ismail, Mervat I. Abd-Elazeem, Emtethal A. Said, Noha M. Khalil, Dina Shahin, Hanan M. El-Saadany, Marwa ElKhalifa, Samah I. Nasef, Ahmed M. Abdalla, Nermeen Noshy, Rasha M. Fawzy, Ehab Saad, Abdelhafeez Moshrif, Amira T. El-Shanawany, Yousra H. Abdel-Fattah, Hossam M. Khalil, Osman Hammam, Aly Ahmed Fathy, Tamer A. Gheita

**Affiliations:** 1 Department of Rheumatology and Rehabilitation, Faculty of Medicine, Assiut University, Assiut, Egypt; 2 Computer Science Department, Higher Institute of Computer Science and Information Systems, Culture and Science City, Giza, Egypt; 3 Ophthalmology Department, Faculty of Medicine, Alexandria University, Alexandria, Egypt; 4 Rheumatology Department, Faculty of Medicine, Cairo University, Cairo, Egypt; 5 Rheumatology Unit, Internal Medicine Department, Faculty of Medicine, Ain-Shams University, Cairo, Egypt; 6 Rheumatology Unit, Internal Medicine Department, Tanta University, Gharbia, Egypt; 7 Rheumatology Unit, Internal Medicine Department, Menoufia University, Menoufia, Egypt; 8 Rheumatology Department, Faculty of Medicine, Zagazig University, Sharkia, Egypt; 9 Rheumatology Unit, Internal Medicine Department, Faculty of Medicine, Beni-Suef University, Beni-Suef, Egypt; 10 Rheumatology Department, Faculty of Medicine, Ain Shams University, Cairo, Egypt; 11 Internal Medicine Department, Rheumatology and Rehabilitation Clinic, National Centre for Radiation Research and Technology, Egyptian Atomic Energy Authority (AEA), Cairo, Egypt; 12 Rheumatology Department, Faculty of Medicine, Sohag University, Sohag, Egypt; 13 Rheumatology Department, Faculty of Medicine, Fayoum University, Fayoum, Egypt; 14 Rheumatology Department, Faculty of Medicine, Mansoura University, Dakahlia, Egypt; 15 Rheumatology Department, Faculty of Medicine, Minia University, Minia, Egypt; 16 Rheumatology Department, Faculty of Medicine, Beni-Suef University, Beni-Suef, Egypt; 17 Rheumatology Department, Faculty of Medicine, Benha University, Kalubia, Egypt; 18 Rheumatology Unit, Internal Medicine Department, Faculty of Medicine, Cairo University, Cairo, Egypt; 19 Rheumatology Unit, Internal Medicine Department, Faculty of Medicine, Mansoura University, Dakahlia, Egypt; 20 Rheumatology Department, Faculty of Medicine, Tanta University, Tanta, Egypt; 21 Rheumatology Unit, Internal Medicine Department, Faculty of Medicine, Alexandria University, Alexandria, Egypt; 22 Rheumatology and Rehabilitation Department, Faculty of Medicine, Suez-Canal University, Ismailia, Egypt; 23 Rheumatology Department, Faculty of Medicine, Aswan University, Aswan, Egypt; 24 Rheumatology Department, Faculty of Medicine, South Valley University, Qena, Egypt; 25 Rheumatology Department, Faculty of Medicine, Al-Azhar University, Assuit, Egypt; 26 Rheumatology Department, Faculty of Medicine, Menoufia University, Menoufia, Egypt; 27 Rheumatology Department, Faculty of Medicine, Alexandria University, Alexandria, Egypt; 28 Ophthalmology Department, Faculty of Medicine, Beni-Suef University, Beni-Suef, Egypt; 29 Department of Rheumatology and Rehabilitation, Faculty of Medicine, New Valley University, New Valley, Egypt; 30 Ophthalmology Department, Faculty of Medicine, Al-Azhar Assiut University, Assiut, Egypt; 31 Rheumatology Department, Kasr Al Ainy School of Medicine, Cairo University, Cairo, Egypt

**Keywords:** Behçet’s disease, Vision-threatening BD, Machine learning, SHAP analysis

## Abstract

**Background:**

Eye lesions, occur in nearly half of patients with Behçet’s Disease (BD), can lead to irreversible damage and vision loss; however, limited studies are available on identifying risk factors for the development of vision-threatening BD (VTBD). Using an Egyptian college of rheumatology (ECR)-BD, a national cohort of BD patients, we examined the performance of machine-learning (ML) models in predicting VTBD compared to logistic regression (LR) analysis. We identified the risk factors for the development of VTBD.

**Methods:**

Patients with complete ocular data were included. VTBD was determined by the presence of any retinal disease, optic nerve involvement, or occurrence of blindness. Various ML-models were developed and examined for VTBD prediction. The Shapley additive explanation value was used for the interpretability of the predictors.

**Results:**

A total of 1094 BD patients [71.5% were men, mean ± SD age 36.1 ± 10 years] were included. 549 (50.2%) individuals had VTBD. Extreme Gradient Boosting was the best-performing ML model (AUROC 0.85, 95% CI 0.81, 0.90) compared with logistic regression (AUROC 0.64, 95%CI 0.58, 0.71). Higher disease activity, thrombocytosis, ever smoking, and daily steroid dose were the top factors associated with VTBD.

**Conclusions:**

Using information obtained in the clinical settings, the Extreme Gradient Boosting identified patients at higher risk of VTBD better than the conventional statistical method. Further longitudinal studies to evaluate the clinical utility of the proposed prediction model are needed.

**Supplementary Information:**

The online version contains supplementary material available at 10.1186/s12911-023-02130-6.

## Background

Behçet's disease (BD) is a chronic systemic immune-mediated vasculitis of unknown cause. Major manifestations include oral and genital ulcers, skin and ocular lesions [1]. Ocular lesions, occur in nearly 48–75% of BD patients [2], are characterized by iridocyclitis, vitritis, retinitis, occlusive retinal vasculitis, and optic disc edema. Poor visual outcome as a result of irreversible ischemic damage of the retina, and optic disc, commonly leads to vision threatening complications in BD (VTBD). Despite available treatment, the rate of poor visual acuity is reported in more than one-third of BD patients [3], and about a quarter of BD patients become blind [4, 5]. According to the BD damage index (BDI), ocular domain represents the top organ contributed to the total BDI score [6]. Accurate identification of patients with BD at risk for vision threatening complications allows initiation of effective treatments to prevent ocular morbidity and save the sight.

Few studies have reported the factors associated with poor ocular outcomes in patients with BD [3, 7, 8]. Higher frequency and longer duration of ocular attacks (uveitis and retinal vasculitis) were among the risk factors for poor visual outcomes and blindness [3, 8]. On the other hand, the presence of systemic vasculitis and genital ulcer was found to be negatively associated with the development VTBD [7]. These findings were limited in their clinical utility by the inclusion of only ocular related variables, the small numbers of patients enrolled, and focusing only on a particular group of BD patients. Prior studies used conventional analysis for the prediction which lack the ability to capture complex interactions among multiple predictors which may limit their use.

To overcome conventional statistical methods’ limitations, machine learning (ML), data analysis technique that develops algorithms by “learning” from data, hold the promise to improve patient classification, predict outcome, and treatment response [9–12]. The ML-based approach was used for diagnosing BD [13], and classification of specific features in patients with BD [14, 15]. Focusing on ocular involvement, ML using multinomial logistic regression was used to determine the misclassification rate of BD-uveitis among 1012 cases of panuveitis [15]. The overall accuracy for BD-panuveitis was 96.3% and 94.0% in the training and validation set respectively.

Given the importance of identifying patients at risk for development of VTBD and no enough data exist, we aimed to develop machine learning models using Egyptian College of Rheumatology (ECR)-BD cohort to predict VTBD and to compare their performance with that of logistic regression. Then, we examined the clinical importance and directionality of each factor for predicting the VTBD using the best performing ML model.

## Methods

### Patients

This population data were derived from ECR-BD cohort, a national study group was created by specialized rheumatologists representing 26 specialized rheumatology centers from 15 major governates around the country from north to south during 2017–2018 [16].

The original ECR-BD database included 1526 adult BD patients (new and existing cases). Inclusion criteria were adults (age ≥ 18 years old) satisfying the diagnostic criteria published by the International Study Group for Behçet’s Disease [17] who presented to one of the included centers. Any patients with other  autoimmune diseases or vasculitis rather than BD or subjects without available ocular outcome data (N = 477) were excluded. This study was approved by the Institutional Review Board of Cairo University, Cairo, Egypt (IRB No.: 47-SReC-RCU2021) and the informed consent was obtained from all participants for the original ECR-BD study [16] and the subsequent secondary data analysis.

### Variables (features) and outcome

#### Predictors (features)

Data were collected on a standard sheet and stored in an electronic database. Patients were subjected to full history taking, clinical examination, and skin pathergy test if required. Medications received by the patients at the time of enrollment were recorded. Disease activity was assessed using the Behçet Disease Current Activity Form (BDCAF) score [14], and laboratory markers were determined for all patients. The data used for this study were fully anonymized. All methods were carried out in accordance with relevant guideline and regulations. All variables reflect patients' status at the time of data entry.

#### Outcome (target) variable

Presence of VTBD (active state at the time of data entry) was diagnosed by ophthalmologists. The full ophthalmological examination included examination of anterior segment by slit lamp, and posterior segment by indirect ophthalmoscopy were conducted for all patients. Fundus fluorescein angiography was done only if needed to confirm posterior segment findings. Patients with retinal disease and/or optic nerve involvement and/or occurrence of blindness were classified as having a VTBD. While, patients with any other form of ocular disease (i.e., episcleritis, cataract, anterior uveitis) were identified as having a non-vision threatening form of the disease (non-VTBD).

#### Supervised ML approaches

ML analyses involved the following steps: data pre-processing, variables (features) selection, model creation, model evaluation, feature importance derivation, and interpretation of results (a positive or negative relationship of each variable with the target variable).

### Data pre-processing and features selection

Data has an overall good quality as the cleaning and transformation process has been made in the primary project [16]. Then, variables of relevance were selected based on clinical expertise, data availability in the database, and literature review. Variables containing a high proportion of missing values (> 30%) were excluded from the analysis. For the included variables with some data missing (< 30%), we applied two techniques; (1) features with missing values were included in the analyses as their own without imputation, and (2) the numerical variables were filled with the mean, and the categorical variables were filled with the mode. Twenty-six variables, which were routinely and easily measured in the clinical setting, were included as inputs to the models (Table 1).

**Table 1:** List of features that were used to building the machine learning algorithms

Sex (male/female)
Age, years
Age at disease onset, years
Disease duration, years
Smoking status (never vs current/former)
Comorbidities (DM, HTN)
Oral ulcer (Y/N)
Genital ulcer (Y/N)
Mucocutaneous involvement (Y/N)
Musculoskeletal involvement (Y/N)
Neurological involvement (Y/N)
Vascular involvement (Y/N)
Gastrointestinal involvement (Y/N)
Disease activity (BDCAF)
Current treatment use (colchicine, MTX, AZA, CYC, CsA, chlorambucil, anticoagulants, biologics) (Y/N)
Current glucocorticoid dose, mg/day
C-reactive protein (CRP), mg/L
Erythrocyte sedimentation rate (ESR), mm/1st hour
Thrombocytosis (Y/N)

Define variables: mucocutaneous involvement includes presence of any erythema, papulopustular, pseudo folliculitis or positive Pathergy test; musculoskeletal involvement includes presence of arthralgia or arthritis, neurological involvement includes stroke, transient ischemic attacks, convulsion, ataxia, cranial or peripheral neuropathy, or psychosis, vascular involvement includes presence of vasculitis, arterial or venous thrombosis, thrombophlebitis, or aneurysm, and gastrointestinal involvement includes presence of diarrhea, bloody diarrhea, or bleeding per rectum

BDCAF, Behçet’s disease current activity form; MTX, methotrexate; AZA, azathioprine; CYC, cyclophosphamide; CsA, cyclosporine A

### Data partition (train and test data)

The total dataset contained 1049 subjects. Prior to training of the algorithms, the data were first split into a training (80%, N = 840) and a test set (20%, N = 209) using a random split. Each model was trained using the training set and evaluated on the test set (i.e., patients not previously seen by the model).

### ML models development

Various ML methods, including extreme gradient boosting (XGBoost) [18], extra Tree Classifier, random forest (RF) [19], support vector machine (SVM), artificial neural networks (ANNs), and multi-layer perceptron (MLP) were applied to classify patients into categories associated with having VTBD. These models are commonly used for binary classification problems in medicine, and to ensure that best possible ML model will be selected. Each model was feeded with the same input variables. A brief summary of the models is presented below:

*Extreme gradient boosting machine* is an ensemble tree-based ML method that includes a chain of classification and regression trees, with each tree created to predict the outcomes misclassified by the previous tree. Thus, the gradient boosting process focuses on predicting more difficult cases and corrects its own weakness. This “boosting” process continues using repeated cross validation, and an ensemble was included to improve robustness when applied to an external dataset. *Extra Tree classifier* generates randomized multiple decision trees with different sub-samples without bootstrapping. It avoids the problem of over-fitting and results in better accuracy. Compared to random forest, extra tree classifier randomly choose the attributes and split the values for tree construction. *Random forest * utilizes multiple classification and regression trees to generate a mean prediction model of case status based on variable importance. When fitting a tree, the random forest algorithm considers a random subset of the predictors at each node and iteratively identifies optimal splits to separate the outcome into two groups with the least disparate outcome probabilities. Random forest accounts for interactions and nonlinear relationships among a large group of factors simultaneously to determine the importance of individual variables, and potentially increasing prediction accuracy. *Support vector machine* is the large margin classifier which classifies the positive and negative data points with a large boundary between them. SVM classifier does not suffer from overfitting problem unlike other similar classifiers. *Artificial neural networks*, non-linear models, consist of a series of layers: the input layer (features), a hidden layer, and an output layer (outcome). Each layer is composed of several units called neurons whose value depends on the connections with the other neurons. *Multi-layer perceptron* is a feedforward artificial neural network that maps input data sets to a set of appropriate outputs. An MLP consists of multiple layers and each layer is fully connected to the next one. The nodes of the layers are neurons with nonlinear activation functions. Between the input and the output layers there may be one or more nonlinear hidden layers.

### Logistic regression

A logistic regression model, representing the simplest of all conventional classifiers, was chosen to create a reference model against the performance of other machine models.

### Models evaluation

In order to evaluate and select the most accurate model, we used the receiver operating characteristic curve (AUROC) (95% confidence intervals (CI)), maximizing AUCROC indicates a satisfying classification. Additionally, the performance of the models was evaluated by accuracy, sensitivity, specificity, negative predictive value (NPV), and positive predictive value (PPV) of each model. We compared models using these criteria of both training and test datasets.

To validate the best models’ results, we used nested K-fold validation in both RF and XGBoost models. Nested K-fold validation ensures our model doesn’t overfit on the training set. The process divided the dataset into five independent folds, and the model is successively trained on four folds and evaluated on the last fold. The evaluation fold rotates so that the process outputs 5 different AUROC. The highest level of accuracy was selected.

### Feature importance

Finally, the interpretation of results in the classifications was evaluated SHapley Additive exPlanation (SHAP) [20]. The SHAP provides the importance and direction of each variable contributing to the model. We present this analysis using SHAP summary and feature importance plots as a method of visual representation.

### Statistical analysis

All data analyses were conducted using Stata statistical software version 15 (Stata-Corp), and Python language (ver.3.7.12). Normally distributed variables were summarized using the mean ± standard deviation (SD), and non-normally distributed variables by the median and interquartile range (IQR). Frequencies were expressed by percentage. Mean characteristics between patients with and without vision threatening complications were compared using a two-sample t-test, and proportions were compared using the chi-square test. Two-sided *P* < 0.05 were considered statistically significant. The SHAP analysis was performed on the cohort subdivided by gender to identify the difference in the affecting factors to the VTBD.

**Fig. 1:**
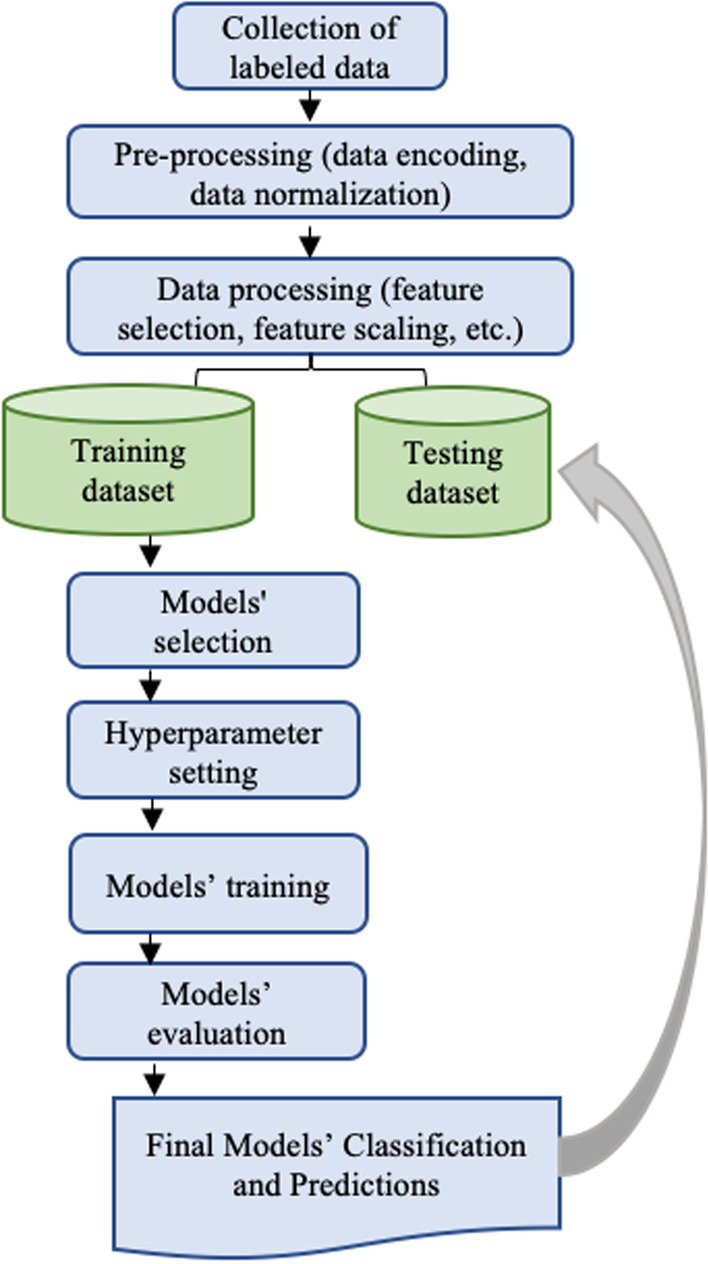
Schematic presentation of the main path of machine learning process used in the current study

For the included variables with some data missing (< 30%), we applied two techniques; (1) features with missing values were included in the analyses as their own without imputation, and (2) missing values were imputed to avoid removing important variables from the dataset. Imputation followed this strategy: in binary variables missing values were substituted by the mode of each class, while in numeric features they were substituted by the mean of each class, a widely used technique [21]. The dictionary contains the scripts, input and output variables for the different analysis included in the manuscript is provided in the following link (https://github.com/aly202012/Beh-et-s-disease-with-Machine-Learning). Schematic presentation of the main process of the machine learning path presents in Fig. 1.

## Results

### Characteristics of the patients’ cohort

In all, 1049 BD patients were analyzed. Among the participants, the mean ± SD age was 36.1 ± 10 years; 71.5% were male; and mean disease duration was 6.7 ± 4.9 years. The mean BDCAF was 4.9 ± 4.5. There were 393 (42.0%) who were smokers, mostly males. 92.1% of the patients were receiving systemic steroids, 83.5% colchicine, 40.7% azathioprine, 26.8% cyclosporine A, 19.4% cyclophosphamide, 7.1% methotrexate, and 7.8% were receiving biologic therapy. 158 (15.7%) were receiving anticoagulants. The main clinical, laboratory and therapeutic features of the whole cohort were described in Table 2.

**Table 2:** Characteristics of Bechet’s disease patients according to the vision threatening complications

Variable Mean ± SD or median (IQR) or N (%)	Total BD patients (n = 1094)	Non-VTBD patients (n = 545)	VTBD patients (n = 549)	*P* value
*Demographic features*				
Age categories				
< 30 years	309 (28.2)	178 (32.7)	131 (23.9)	< 0.0001
30–40 years	449 (41.0)	228 (41.8)	221 (40.3)	
> 40 years	336 (30.7)	139 (25.5)	197 (35.9)	
Disease duration				0.552
≤ 5 years	486 (44.4)	247 (45.3)	239 (43.5)	
> 5 years	608 (55.6)	298 (54.7)	310 (56.5)	
Age at onset				0.465
< 40 years	570 (52.1)	290 (53.2)	280 (51.0)	
≥ 40 years	524 (47.9)	255 (46.8)	269 (49.0)	
Male gender	782 (71.5)	399 (73.2)	383 (69.8)	0.207
Body mass index	28.0 ± 5.5	27.5 ± 5.3	28.8 ± 5.8	0.067
Smoking status				0.033
Never	543 (58.0)	269 (61.7)	274 (54.8)	
Ever smoker	393(42.0)	167 (38.3)	226 (45.2)	
*Clinical features, N (%)*				
Oral ulcers	1094 (100)	454 (100)	549 (100)	–
Genital ulcers	903(83.4)	460 (85.3)	443 (81.4)	0.084
Mucocutaneous manifestations	586 (58.5)	276 (55.9)	310 (61.0)	0.098
Ocular manifestations	812 (78.0)	304 (59.6)	508 (95.7)	< 0.0001
Musculoskeletal manifestations	361(34.6)	148 (28.6)	213 (40.5)	< 0.0001
Neurological manifestations	159 (14.9)	63 (11.8)	96 (18.0)	0.005
Vascular manifestations	239 (24.5)	120 (25.3)	119 (23.8)	0.608
Gastrointestinal manifestations	104 (10.6)	42 (8.8)	62 (12.4)	0.065
BDCAF	4.9 ± 4.5	5.0 ± 3.8	4.8 ± 4.9	0.649
Diabetes mellitus	204 (21.4)	75 (15.9)	129 (26.9)	< 0.0001
Hypertension	265 (27.5)	85 (17.7)	180 (37.3)	< 0.0001
*Treatments, N(%)*				
Colchicine	517 (83.5)	336 (85.3)	181 (80.4)	0.119
Steroid dose (mg/day)	1 (0, 62.0)	1 (0, 45.1)	1 (1, 41.5)	0.867
Steroid use	855 (92.1)	379 (84.8)	476 (99.0)	< 0.0001
Cyclosporine	273 (26.8)	89 (17.5)	184 (36.1)	< 0.0001
AZA	429 (40.7)	266 (50.5)	163 (30.9)	< 0.0001
CYC	194 (19.4)	105 (21.4)	89 (17.5)	0.115
Chlorambucil	8 (0.84)	0 (0.0)	7 (1.4)	–
Anticoagulant	158 (15.7)	104 (20.9)	54 (10.6)	< 0.0001
MTX	65 (7.1)	43 (9.7)	22 (4.7)	0.003
Biologics	76 (7.8)	21 (4.5)	55 (11.0)	< 0.0001
*Laboratory manifestations*				
HGB (g/dl)	12.8 ± 1.7	12.6 ± 1.7	13.3 ± 1.4	< 0.0001
TLC (× 10^3^/mm^3^)	8.0 ± 3.1	8.0 ± 3.2	8.1 ± 2.9	0.860
PLT (× 10^3^/mm^3^)	263.9 ± 80.5	272.0 ± 86.5	250.5 ± 67.8	0.001
ESR (mm/1sthr)	30.0 ± 20.5	31.1 ± 20.9	29.0 ± 20.1	0.107
CRP titre (mg/L)	8 (0, 96)	9.2 (0, 96)	6.9 (0, 55.3)	< 0.0001
SUA	4.8 ± 1.6	4.7 ± 1.4	5.0 ± 1.4	0.247

BDCAF, Behçets disease current activity form; HGB, hemoglobin; TLC, total leucocyte count; PLT, platelet count; ESR, erythrocyte sedimentation rate; CR, C-reactive protein; SUA, serum uric acid

Overall 78.0% of patients had any form of ocular involvements.Vision threatening manifestations were identified in 549 (50.2%) patients. Most frequent ocular manifestations were anterior uveitis in 542 (50.2%), posterior uveitis in 575 (53.6%), retinal occlusion in 273 (28.7%), cataract and conjunctivitis in 318 (41.9%), optic nerve involvement in 106 (24.1%), and 37 (7.9%) patients were blind.

### Performance of various prediction models for predicting VTBD

Table 3 and Additional file 1: Figure S1 summarize the discrimination performance of the models. In all models, the AUCROC for predicting VTBD ranged from 0.64 to 0.85. The AUCROC of the conventional logistic regression model was 0.64 (95% CI 0.58, 0.71). On the other hand, XGBoost showed the best performance ML model in predicting VTBD with an overall AUROC of 0.85 (95% CI 0.81, 0.90). The specificity, sensitivity, and accuracy for this model were 0.86, 0.85, and 0.85, respectively. Random forest performance followed XGBoost model (AUROC = 0.83, 95% CI 0.79, 0.89). Then using K-fold validation method, the highest accuracy was 84.0% for RF and 83.1% for XGBoost models.

**Table 3:** Results of the model performance on training and test sets

	Training set (N = 840)						Test set (N = 209)					
	AUROC (95%CI)	Accuracy	Sensitivity	Specificity	PPV	NPV	AUROC (95%CI)	Accuracy	Sensitivity	Specificity	PPV	NPV
XGBoost	0.98 (0.97,0.99)	0.79	0.99	0.98	0.97	0.99	0.85 (0.81,0.90)	0.85	0.85	0.86	0.86	0.84
RF	0.99 (0.98,0.99)	0.99	0.99	0.99	0.99	0.99	0.83 (0.79,0.89)	0.83	0.83	0.84	0.84	0.82
Extra tree	0.99 (0.98,0.99)	0.99	0.98	0.99	0.99	0.98	0.79 (0.69,0.89)	0.79	0.81	0.77	0.79	0.80
SVM	0.72 (0.69,0.75)	0.73	0.66	0.84	0.89	056	0.78 (0.73,0.83)	0.78	0.73	0.86	0.90	0.66
ANN	0.66 (0.62,0.69)	0.57	0.64	0.56	0.35	0.81	0.67 (0.61,0.73)	0.67	0.68	0.66	0.66	0.67
MLP	0.72 (0.67,0.78)	0.70	0.71	0.69	0.67	0.73	0.66 (0.59,0.72)	0.66	0.71	0.63	0.57	0.76
LR	0.67 (0.63,0.69)	0.67	0.72	0.63	0.55	0.78	0.64 (0.58,0.71)	0.65	0.75	0.60	0.46	0.84

Models are listed in order of decreasing AUROC

AUROC, area under the curve; XGBoost, extreme gradient boosting; RF, random forest; SVM, support vector machine; ANN, artificial neural networks; MLP, multi-layer perceptron; LR, logistic regression; PPV, positive predictive value; NPV, negative predictive value

### Feature importance and model interpretation

**Fig. 2:**
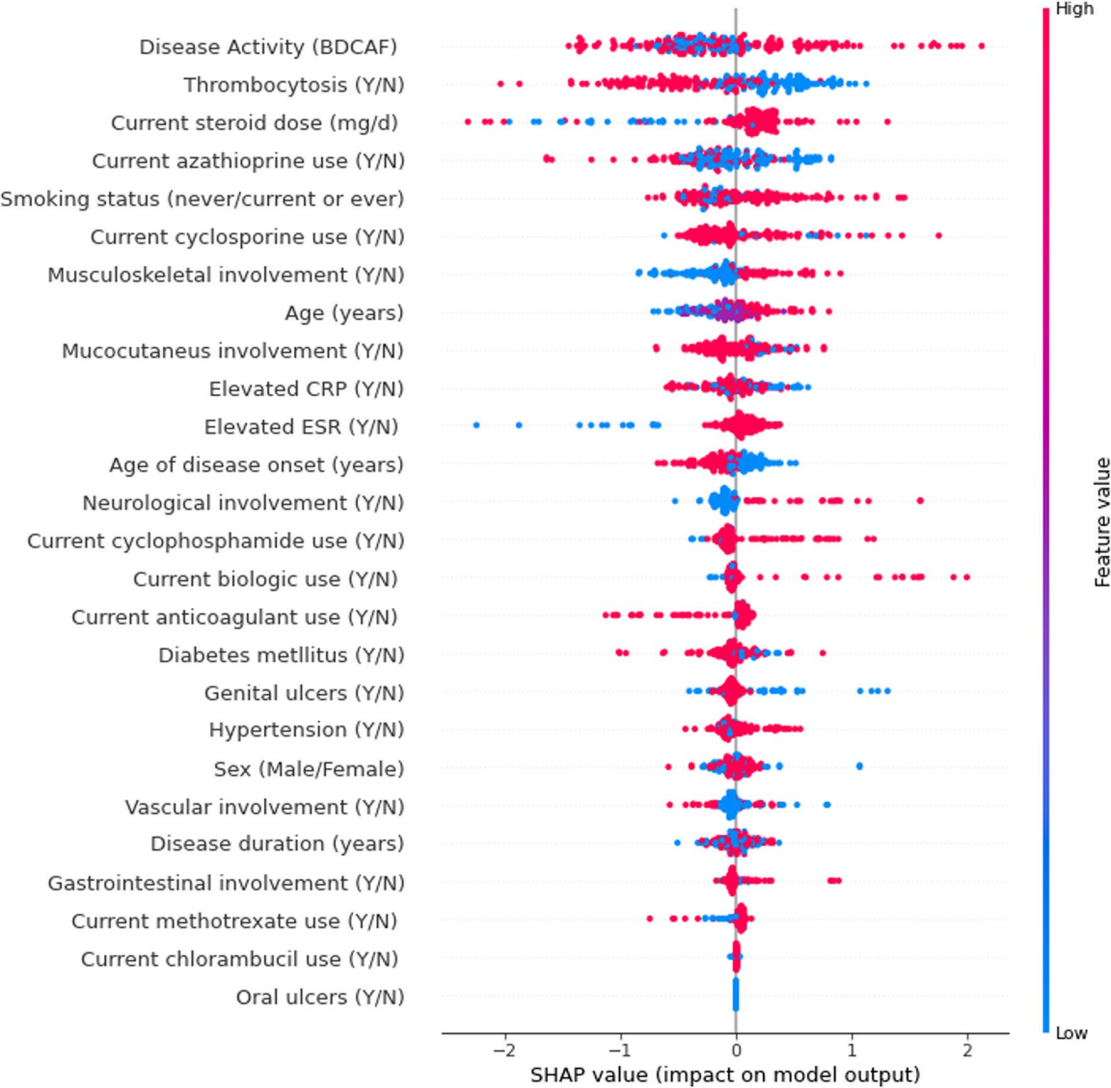
Overall SHAP values for the variables in Shapely plots to display both the feature importance and feature contribution to the model prediction. Shapley plots show the SHAP values in the order of the important variables that contribute to VTBD. The x-axis represents the marginal contribution of a feature to the change in the predicted probability of development of VTBD. Colors indicate the value of the variable: red represents higher numerical values of the variable and blue represent lower numerical values. As all categorical variables were converted into binary indicators, zero (i.e., absence) is indicated with blue dots and one (i.e., presence) is represented by red dots

Figure 2 shows the influence of variables to VTBD in the prediction model. Disease activity, thrombocytosis, smoking status, and daily steroid dose were among the top estimators for the presence of VTBD. On the other hand, oral ulcers, chlorambucil and methotrexate use, and gastrointestinal involvement were less important factors compared to the other selected features.

In term of interpretation of the SHAP dots, for example, the blue (lighter) colors for disease activity (BDCAF) represent lower values, whereas the red (darker) colors represent higher values. Positive SHAP values with red colors indicate that higher BDCAF values related to VTBD, whereas negative SHAP values with lighter colors indicate that lower BDCAF values are strongly protective for presence of VTBD. For the categorical features, patients with musculoskeletal manifestations were more likely to have VTBD as suggested by the larger spread of the red dots on the right.

**Fig. 3:**
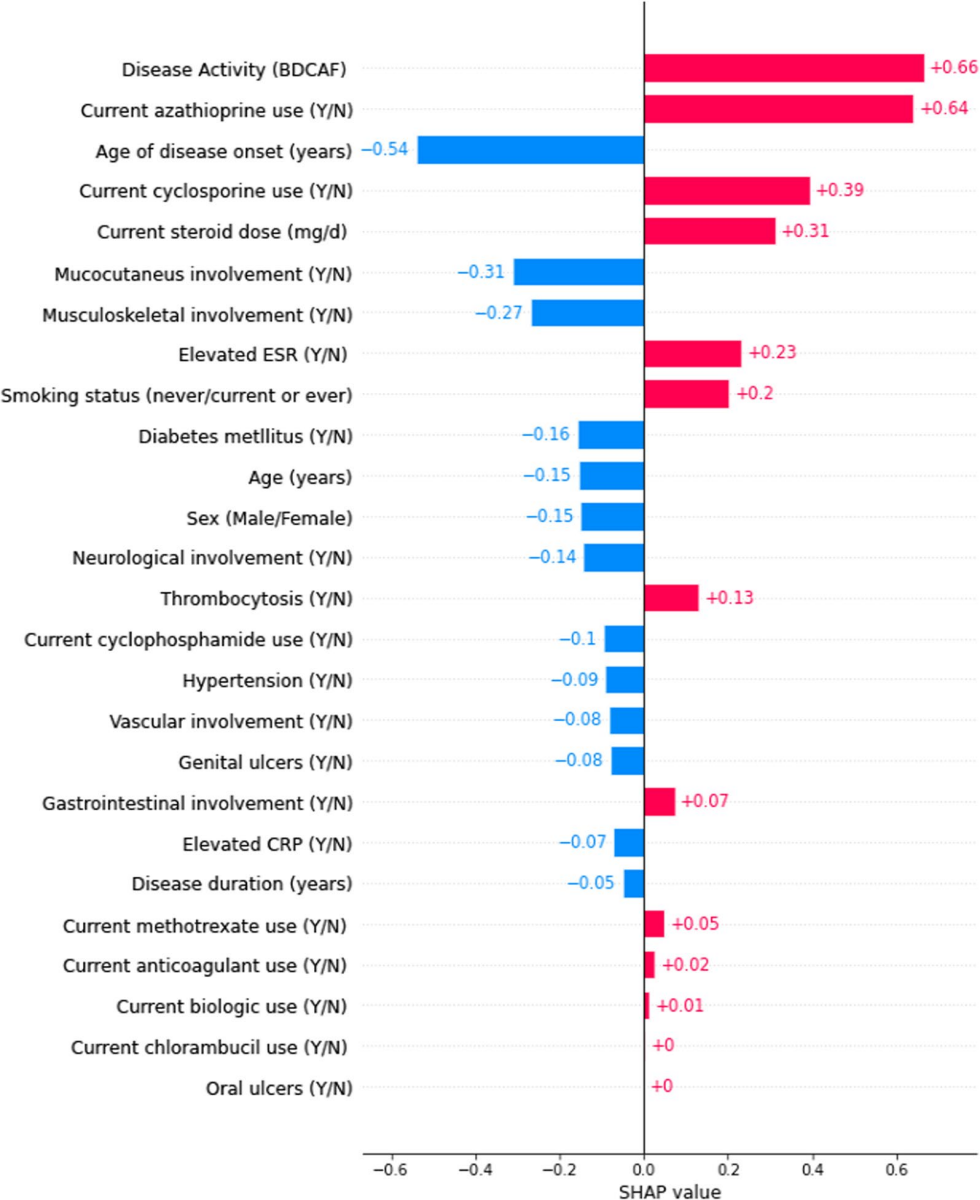
XGBoost variable importance for predicting VTBD. The x-axis shows how much each feature added or subtracted to the final probability value for VTBD development. Please note that the numbers presented are average contributions for each feature to the model prediction

The associations and average contributions of these features to the absolute predicted probability of VTBD are presented in Fig. 3. BDCAF was the most important predictor and it increased the absolute values of the predicted probability of VTBD by an average of 0.66. Both high ESR and thrombocytosis were associated with a small but positive increase in the predicted probability of potentially VTBD (0.23 and 0.13; respectively). The younger age of disease onset was associated with lower predicted probabilities of existence of VTBD (− 0.54).

### Important variables for predicting VTBD stratified by gender

**Fig. 4:**
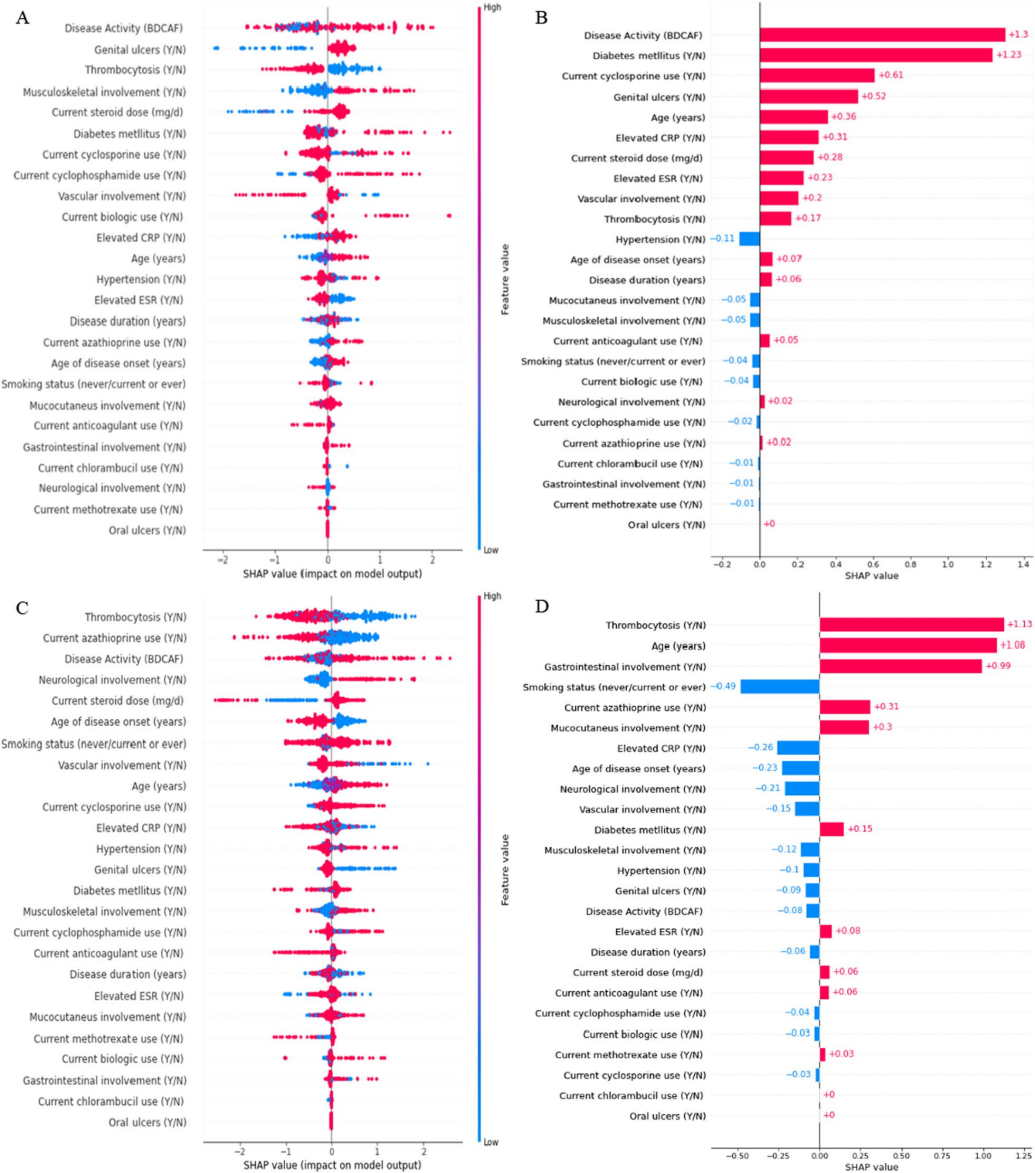
XGBoost variable importance and overall SHAP values for predicting VTBD among women and men with BD. Figure shows degree and direction of contribution of the variables to VTBD from the Shapley plot separated by gender. Shapley plots show the SHAP values in the order of the important variables that contribute to VTBD (left side) and by the direction of the contribution (right side). **A** Represents the variables importance in women, while **B** shows the average probability value of each variables in the contribution of VTBD in women. **C** Represents the variables importance and **D** shows the average probability value of each variables in the contribution of VTBD in men

The basic characteristics of the cohort stratified by gender are presented in Additional file 2: Table S1. Figure 4 shows degree and direction of contribution of the variables to VTBD from the Shapley plot separated by gender. In women with BD; BDCAF score, genital ulcer, thrombocytosis, and musculoskeletal manifestations were the top variables that contribute to the VTBD model (Fig. 4A). Higher BDCAF score and presence of diabetes mellitus were associated with higher probability of VTBD (Fig. 4B). For men with BD; thrombocytosis, older age, and presence of gastrointestinal manifestations were associated with a higher probability of VTBD (Fig. 4C). Contradictory to women, genital ulcers were associated with less probability for VTBD in men indicated by more red dots on the left (SHAP values − 0.09) as shown in Fig. 4D.

## Discussion

Predicting the outcome of ocular disorders in patients with BD, and providing proper and effective care for these patients can improve the visual acuity prognosis. We used a machine learning approach for predicting this severe complication selection, which is suited to data with high dimensionality. In this study, machine learning approaches outperformed the traditional statistical methods in the detection of vision threatening complications among patients with BD. In addition, we identified the important factors that were associated with VTBD risk. An advantage of this approach is that it builds on basic predictors available in the routine setting making the prediction model easily implemented in clinical practice. Further reproducible studies to validate these exploratory ML models in a larger number of BD patients are needed.

Machine learning is more suitable than conventional statistical approaches when: (1) there is no great prior knowledge on the topic under  investigation; and (2) the number of observations largely exceeds the allowed number of input variables [22]. The study demonstrates that XGBoost, the best performing ML mode (accuracy 85.0%) was superior to LR (accuracy 64.0%), which suggests that the relationship between input variables and the predictive outcome is nonlinear. This is in line with previous studies in the field of prediction task of medicine [10, 23, 24]. Although, there are no other studies related to developing ML algorithms for detecting the visual outcome of BD-affected patients, researchers successfully applied ML for a range of BD manifestations’ discrimination [14, 15]. For example, the application of ML classification to discriminate 194 cases of BD with uveitis against other inflammatory uveitis showed an overall accuracy for panuveitides was 96.3% in the training set and 94.0% in the validation set [15]. Using K-fold validation, the XGBoost model recorded a slightly lower level of accuracy than RF (83.1% vs 84.0%). This result can be explained by the small size of the data in each fold when the model builds the tree that records a higher level of accuracy compared to the previous fold. The data in the Fold itself does not contain properties that can be used to build the most appropriate tree until the model exceeds the specified accuracy level.

As opposed to the “black-box” problem of ML, the feature analysis based on the Shapley values, was recently applied  to improve the interpretability of predicting complex models [25]. The current study was identified that thrombocytosis among the important factors contribute to presence of VTBD. Previous research has shown the potential role of platelet either the count parameter (thrombocytosis) or function parameter (mean platelet volume) in BD patients [26]. Although the underlying pathophysiology is not well-understood, platelets have a central role in the pathogenesis of thrombosis. BD is characterized by the venous as well as arterial thrombosis [27]. As platelet counts measurement is low in costs, and readily available in the clinical settings, they could be valuable potential markers in the evaluation of ocular disorders progression.

Interestingly, the feature importance analysis shows an important role for BDCAF score in the estimation of VTBD; higher BDCAF score is associated with a higher probability for VTBD. Disease activity is the presence of any ongoing expression of vasculitis that may precede the disease damage [28]. There was no significant correlation between BDCAF score with the total disease damage assessed either by BDI or vasculitis damage index [6, 29]. A prospective study measures BDCAF score over longitudinal period is preferred to examine the impact of disease activity on the development of VTBD.

This work shows that genital ulcer, systemic vasculitis, and oral ulcers had the lowest probability to be associated with VTBD. Among 249 subjects with BD, these three systemic factors have a predictive value on the development of non-VTBD, defined as a milder form of eye involvement [7]. In other studies, local ocular factors: such as higher frequency of ocular attacks and longer duration of uveitis and retinal vasculitis were the main risk factors for blindness among patients with BD [3, 8]. Although, traditional statistical approaches have suggested some associations, no previous work from a high quality, large dataset using wide spectrum of variables collected in a clinical setting was determined.

There are well-known gender differences in the clinical manifestations and severity of BD [16, 30]; however, few data are available concerning gender difference in the BD associated ocular disorders and their predictors. Different variables were identified as the top features for detecting VTBD in both sexes. For men, thrombocytosis, older age, and gastrointestinal involvement were the top features associated with VTBD, while, in women, higher BDCAF, presence of genital ulcers, and musculoskeletal involvement were the important features for VTBD. Interestingly, genital ulcer was associated with lower predicted probabilities of VTBD in women, but with higher probability of VTBD in men. The course of the BD ocular manifestations is known to be more severe in the male population [30–32]; however, Davatchi et al. [33] have shown that the severity of ocular BD had the same outcome and improvement under treatment in the two sexes. Although the etiology of ocular manifestations is unknown, both genetic and environmental factors (smoking, infection, and vitamin D ) have been blamed [34, 35]. While, smoking was the 4th ranked important feature in men, it was the 17th ranked feature in women associated with less probability of VTBD (Fig. 4). Smoking was more common among male patients with BD in some studies raising the question of possible association [36, 37]. Identification of gender difference risk factors for VTBD may indeed be responsible for more severe disease in men.

Although RF presented a comparable accuracy, the XGBoost was considered the ideal model in terms of the results being easier to interpret, and thus allowing to better understand the factors influencing the prediction result [38]. The random forest model aggregates multiple decision trees grown on bootstrap subsamples of the training set, while the XGBoost successively build decision trees, learning from the mistakes of the previous ones. The XGBoost method often achieve the best results on structured data [39] as available in our study. The XGBoost model presented here, if validated for use, is of great clinical interest because it requires demographic and clinical variables only, with no genetic or biomarkers data. In daily practice, prognostic models that would be available at the time of decision-making are preferred. In this way, the rheumatologist can select patients for recommended ophthalmologic counselling, and can decide the appropriate treatment for these patients.

This study has several strengths. First, we used well-curated data available from national BD cohort to generate our classification models. Second, given that the patients were from multicenter and because the partitions were non-random, this approach is considered a type of validation. Third, the use of ensemble tree-based model was a strength owing to its capability to examine a large number of variables, accounting for all others simultaneously. Finally, we provided a Shapley plot that can be easily explained visually and easily to understand. This study still has some limitations. The cross-sectional design, thus the realization of a longitudinal analysis, is certainly needed. Analyses with a large number of variables are susceptible to collinearity among the variables; however, XGBoost classifier is designed to account for collinearity. Patients with BD were excluded if they were missing outcome data in their databases, which may introduce bias to the analysis. In the current study, there is male predilection (71.5%), which is consistent with previous reports from Middle East (REF). However the analysis were stratified by gender differences, further validation of ML models in different gender distribution dataset is suggested. Finally, the decision about what to do when the value of a patient’s variable was missing was a challenge. Our choice to not replace missing values was supported by the similar results we observed with statistical imputation (Additional file 2: Table S2).

## Conclusion

In conclusion, we identified that Extreme Gradient Boosting model could reliably identify features associated with BDVT risk better than the conventional statistical method. Furthermore, higher disease activity, thrombocytosis, ever smoking, and daily steroid dose were the top factors associated with VTBD. The identification of VTBD-key contributors improve the multimodal treatment strategies. Such approach could be further validated on an external dataset, and once validated, it would be easy to implement at the point of care to individualize and tailor therapeutic regimens.

## Supplementary Information

**Additional file 1.** **Supplementary figure 1:** Receiver operating characteristic (ROC) curve analysis of machine learning algorithms for prediction of VTBD in the training (Left figure) and testing (Right figure) sets.

**Additional file 2.** **Supplementary table 1:** Characteristics of Bechet’s disease patients stratified by gender.

## Data Availability

The datasets generated during and/or analysed during the current study, and the code required for replicating the results in the paper are available upon request on request to the corresponding author.

## Abbreviations

BD: Behçet’s disease
VTBD: Vision threatening complications in BD
BDI: BD damage index
ML: Machine learning
ECR: Egyptian College of Rheumatology
BDCAF: Behçet’s Disease Current Activity Form
XGBoost: Extreme gradient boosting
ETC: Extra tree classifier
RF: Random forest
SVM: Support vector machine
ANNs: Artificial neural networks
MLP: Multi-layer perceptron
AUROC: Receiver operating characteristic curve
CI: Confidence intervals
NPV: Negative predictive value
PPV: Positive predictive value
SHAP: SHapley additive explanation
SD: Standard deviation
IQR: Interquartile range (IQR)
