# Supplementary material for: Development of machine learning models for detection of vision threatening Behçet’s disease (BD) using Egyptian College of Rheumatology (ECR)–BD cohort
Source: BMC Med Inform Decis Mak. 2023 Feb 17;23:37. doi: 10.1186/s12911-023-02130-6 (PMC9938580; doi:10.1186/s12911-023-02130-6)
Supplement: Supplementary file 1 — Additional file 1. Supplementary figure 1: Receiver operating characteristic (ROC) curve analysis of machine learning algorithms for prediction of VTBD in the training (Left figure) and testing (Right figure) sets. [file 12911_2023_2130_MOESM1_ESM.docx]

Supplementary figure 1: Receiver operating characteristic (ROC) curve analysis of machine learning algorithms for prediction of VTBD in the training (Left figure) and testing (Right figure) sets.

**
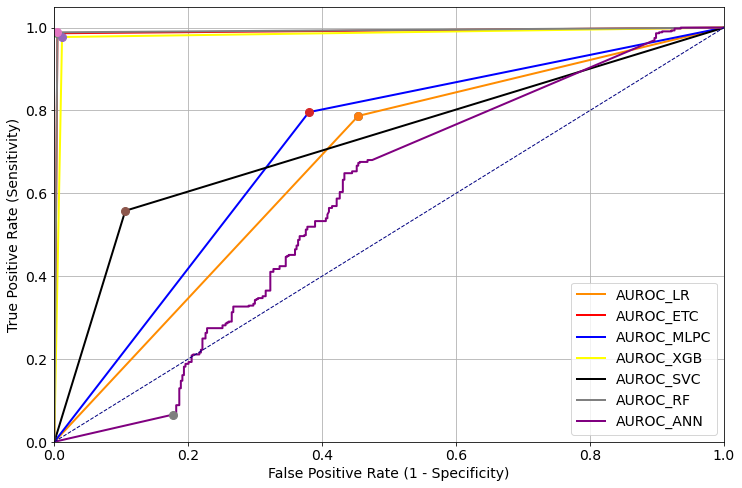
** **
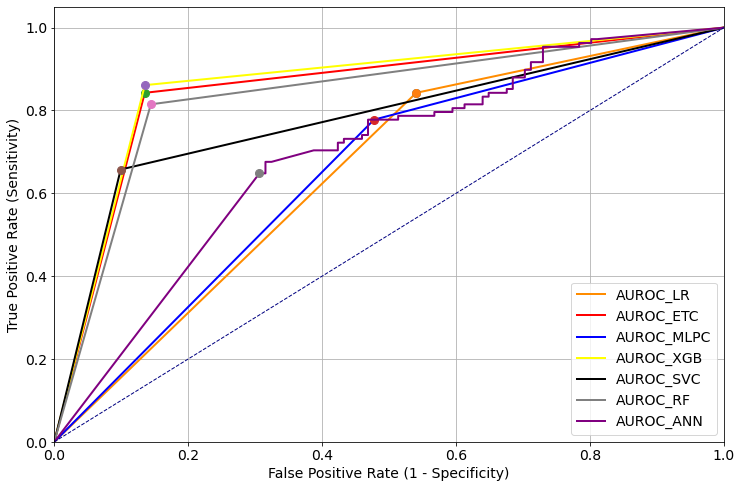
**

LR, Logistic regression; ETC, Extra tree classifier; MLPC, multi-layer perceptron; XGB, extreme gradient boosting; SVC, support vector machine; RF, random forest; and ANN, artificial neural networks (ANNs).
