# Supplementary material for: Development of machine learning models for detection of vision threatening Behçet’s disease (BD) using Egyptian College of Rheumatology (ECR)–BD cohort
Source: BMC Med Inform Decis Mak. 2023 Feb 17;23:37. doi: 10.1186/s12911-023-02130-6 (PMC9938580; doi:10.1186/s12911-023-02130-6)
Supplement: Supplementary file 2 — Additional file 2. Supplementary table 1: Characteristics of Bechet’s disease patients stratified by gender. [file 12911_2023_2130_MOESM2_ESM.docx]

Supplementary table 1: Characteristics of Bechet’s disease patients stratified by gender

| Variable  Mean±SD or median (IQR) or N (%) | Female patients  312 (28.5) | Male patients  782 (71.5) | P value |
| --- | --- | --- | --- |
| Demographic features | | | |
| Age categories  <30 years  30-40 years  >40 years | 93 (29.8)  126 (40.4)  93 (29.8) | 216 (27.6)  323 (41.3)  243 (31.1) | 0.763 |
| Disease duration  ≤5 years  >5 years | 247 (45.3)  298 (54.7) | 239 (43.5)  310 (56.5) | 0.552 |
| Age at onset  <40 years  ≥40 years | 170 (54.5)  142 (45.5) | 400 (51.1)  382 (48.8) | 0.319 |
| Body mass index | 31.1±7.0 | 27.5±5.1 | <0.001 |
| Smoking status  never  ever smoker | 213 (81.6)  48 (18.4) | 330 (48.9)  345 (51.1) | <0.001 |
| Clinical features, N (%) | | | |
| Oral ulcers | 312 (100) | 782 (100) | -- |
| Genital ulcers | 258 (83.2) | 645 (83.4) | 0.931 |
| Mucocutaneous manifestations | 167 (57.0) | 419 (59.1) | 0.539 |
| Ocular manifestations | 241 (79.3) | 571 (77.5) | 0.524 |
| Musculoskeletal manifestations | 118 (39.9) | 243(32.5) | 0.024 |
| Neurological manifestations | 31 (10.2) | 128 (16.8) | 0.007 |
| Vascular manifestations | 53 (18.5) | 186 (27.0) | 0. 005 |
| Gastrointestinal manifestations | 22 (7.7) | 82 (11.9) | 0.051 |
| BDCAF | 5.6±4.7 | 4.5±4.4 | 0.003 |
| Diabetes mellitus | 60 (23.3) | 144 (20.7) | 0.396 |
| Hypertension | 97 (37.4) | 168 (23.9) | <0.0001 |
| Treatments, N(%) | | | |
| Colchicine | 126 (82.9) | 391 (83.7) | 0.810 |
| Steroid dose (mg/day) | 1 (0, 55.0) | 1 (0, 66.7) | 0.613 |
| Steroid use | 257 (91.8) | 598 (92.3) | 0.796 |
| Cyclosporine | 66 (22.7) | 207 (28.4) | 0.067 |
| AZA | 119 (39.4) | 310 (41.2) | 0.587 |
| CYC | 53 (18.5) | 141 (19.8) | 0.629 |
| Chlorambucil | 4 (1.4) | 4 (0.59) | 0.192 |
| Anticoagulant | 29 (10.2) | 129 (17.9) | 0.003 |
| MTX | 21 (8.5) | 44 (6.6) | 0.304 |
| Biologics | 37 (13.1) | 39 (5.7) | <0.0001 |
| Laboratory manifestations | | | |
| HGB (g/dl) | 12.1±1.7 | 13.1±1.6 | <0.0001 |
| TLC (x10^3^/mm^3^) | 7.7±2.8 | 8.1±3.2 | 0.125 |
| PLT (x10^3^/mm^3^) | 277.3±84.9 | 259.6±78.7 | 0.021 |
| ESR (mm/1^st^hr) | 33.8±20.6 | 28.4±20.3 | <0.0001 |
| CRP titre (mg/L) | 12.0 (0, 39.7) | 7.0 (0, 96.0) | 0.631 |
| SUA | 4.1±1.5 | 4.9±1.6 | 0.001 |

BDCAF: Behçets disease current activity form; HGB: hemoglobin; TLC: total leucocyte count; PLT: platelet count; ESR: erythrocyte sedimentation rate; CR: C-reactive protein; and SUA: serum uric acid.

Supplementary table 2: Results of the model performance on training and test sets using the imputation of missing values

|  | Training set (N=840) | | | | | | Test set (N=209) | | | | | | | |
| --- | --- | --- | --- | --- | --- | --- | --- | --- | --- | --- | --- | --- | --- | --- |
|  | AUROC  (95%CI) | Accuracy | Sensitivity | Specificity | PPV | NPV | AUROC  (95%CI) | Accuracy | Sensitivity | | Specificity | PPV | NPV |  |
| XGBoost | 0.98  (0.97,0.99) | 0.98 | 0.97 | 0.99 | 0.99 | 0.97 | 0.83  (0.74,0.85) | 0.83 | 0.82 | 0.83 | | 0.85 | 0.81 |  |
| RF | 0.99  (0.98,0.99) | 0.99 | 0.99 | 0.99 | 0.99 | 0.99 | 0.82  (0.77,0.87) | 0.82 | 0.83 | 0.81 | | 0.81 | 0.83 |  |
| Extra tree | 0.99  (0.98,0.99) | 0.99 | 0.99 | 0.98 | 0.98 | 0.99 | 0.82  (0.77,0.87) | 0.82 | 0.81 | 0.83 | | 0.84 | 0.79 |  |
| SVM | 0.84  (0.82,0.87) | 0.84 | 0.81 | 0.88 | 0.89 | 079 | 0.81  (0.76,0.87) | 0.81 | 0.81 | 0.82 | | 0.82 | 0.80 |  |
| ANN | 0.80  (0.72,0.82) | 0.79 | 0.78 | 0.80 | 0.81 | 0.77 | 0.72  (0.70,0.81) | 0.72 | 0.74 | 0.70 | | 0.69 | 0.75 |  |
| MLP | 0.96  (0.96,0.98) | 0.70 | 0.97 | 0.96 | 0.96 | 0.97 | 0.79  (0.76,0.82) | 0.79 | 0.81 | 0.77 | | 0.76 | 0.81 |  |
| LR | 0.68  (0.65,0.72) | 0.68 | 0.69 | 0.68 | 0.66 | 0.71 | 0.72  (0.65,0.77) | 0.72 | 0.75 | 0.68 | | 0.66 | 0.77 |  |

AUROC: area under the curve; XGBoost: extreme gradient boosting; RF: random forest; SVM: support vector machine; ANN: artificial neural networks; MLP: multi-layer perceptron; LR: logistic regression; PPV: positive predictive value; and NPV: negative predictive value. Models are listed in order of decreasing AUROC.
